# Migration and cognitive function: a conceptual framework for Global Health Research

**DOI:** 10.1186/s41256-018-0088-5

**Published:** 2018-11-22

**Authors:** Hanzhang Xu, Allison A. Vorderstrasse, Eleanor S. McConnell, Matthew E. Dupre, Truls Østbye, Bei Wu

**Affiliations:** 1 School of Nursing, Duke University, Durham, NC USA; 2 Department of Community and Family Medicine, Duke University, Durham, NC USA; 3 New York University Rory Meyers College of Nursing, New York, NY USA; 4 Geriatric Research, Education and Clinical Center, Durham Department of Veterans Affairs Healthcare System, Durham, NC USA; 5 Department of Population Health Sciences, Duke University, Durham, NC USA; 6 Duke Clinical Research Institute, Duke University, Durham, NC USA; 7 Department of Sociology, Duke University, Durham, NC USA; 8 Duke Global Health Institute, Duke University, Durham, NC USA

## Abstract

**Background:**

Migration is a fundamental demographic process that has been observed globally. It is suggested that migration is an issue of global health importance that can have an immediate and lasting impact on an individual’s health and well-being. There is now an increasing body of evidence linking migration with cognitive function in older adults. In this paper, we synthesized the current evidence to develop a general conceptual framework to understand the factors contributing to the association between migration and cognitive function.

**Methods:**

A comprehensive review of the literature was conducted on the associations between migration and cognition among middle-aged and older adults.

**Results:**

Five potential mechanisms were identified from the literature: 1) socioeconomic status—including education, occupation, and income; 2) psychosocial factors—including social networks, social support, social stressors, and discrimination; 3) behavioral factors—including smoking, drinking, and health service utilization; 4) physical and psychological health status—including chronic conditions, physical function, and depression; and 5) environmental factors—including both physical and social environment. Several underlying factors were also identified—including early-life conditions, gender, and genetic factors.

**Conclusions:**

The factors linking migration and cognitive function are multidimensional and complex. This conceptual framework highlights potential implications for global health policies and planning on healthy aging and migrant health. Additional studies are needed to further examine these mechanisms to extend and refine our general conceptual framework.

## Background

Migration is the geographic movement of individuals across a specified boundary for the purpose of establishing a new residence [1]. Migrant populations, both within countries and internationally, have been increasing for the past few decades [2]. According to recent estimates, in 2013, there were more than 232 million international migrants; and another 740 million internal migrants worldwide [2]. This dramatic increase in the migrant populations has drawn particular attention to migration and health and highlights the needs to identify best practices to promote healthy aging for migrant populations. Cognitive impairment, often defined as an individual’s experienced difficulties in remembering things, learning new skills, concentrating on tasks, or making decisions, is a common problem in old age [3]. As these migrant populations get older, many will experience cognitive decline [4–6]. Increasing number of older adults experiencing some level of cognitive decline continue to put enormous strain on healthcare systems and on caregivers who provide care for people with cognitive impairment [6, 7]. Thus, it is important to understand how migration may relate to initial levels of cognitive function and the rate of cognitive change over time [8]. A variety of factors—such as sociodemographic background, health behaviors, and genetic traits—can influence levels of cognitive function and changes over time [9]. Likewise, the migration process is related to many of these factors and, therefore, may be important mechanisms in the association between migration and cognitive function.

The purpose of this paper is to present a general conceptual framework of the linkages between migration and cognitive function. We conduct a comprehensive review of the literature and discuss the possible mechanisms that may explain the association between migration and cognitive function. Furthermore, synthesizing the current evidence to provide a conceptual framework will help elucidate important mechanisms and provide guidance for researchers to develop effective approaches to prevent cognitive decline among older adult populations. In addition, this conceptual framework will highlight potential actionable areas that inform the development of global health policies and planning on healthy aging and migrant health.

## Theories and current literature on migration and cognitive function

Our previous systematic review synthesized the current literature on migration and cognitive function [10]. We found that different migration patterns have different impacts on cognitive function [10]. We also found that there is a lack of a conceptual framework that elucidates the potential pathways between migration and cognitive function in the current literature [10]. Existing theories, such as the push-pull theory that has been widely used by economists, do not explicitly explain how migration might influence an individual’s cognitive status [11, 12]. Given the sheer number of migrants across the globe, it is important to develop a conceptual framework to examine the underlying linkages that connect migration and cognition. Building on our published systematic review, we first reviewed three theories and models that have been used in the current literature related to migration and health.

### Life course perspective

The life course perspective has served as a useful interdisciplinary framework in social, behavioral, and health science research [13–15]. Elder and colleagues describe the life course as “consisting of age-graded patterns that are embedded in social institutions and history” [15]. To illustrate, social and physical exposures during critical periods (e.g. gestation, childhood, and adulthood) may have cumulative effects on health status in later life, such as through increasing the risk of chronic conditions and influencing functional status [16]. In the context of migration and cognitive function, migration may alter an individual’s trajectory of cognitive function. The timing and duration of migration might have an impact on the magnitude of the relationship between migration and cognition. Still, the life course perspective fails to incorporate the determinants of health status—cognitive function in this case—and the reasons for migration. Therefore, other theories and models are needed to address the limitation of life course theory in guiding future research on migration and cognition.

### Social determinants of health

The World Health Organization first introduced the concept “determinants of health” to illustrate the idea that an individual’s health status is determined by many factors together [17]. Based on the social determinants of health model, factors that influence health status include but are not limited to 1) the social and economic environment (e.g. income, education), 2) the physical environment (e.g. clean water, safe housing), and 3) the individual’s characteristics and behaviors (e.g. access to healthcare, smoking). The social determinants of health model has been widely embedded in studies related to disease and functional status [18–20]. A recent study has comprehensively summarized the key risks and protective factors related to cognitive impairment [9]. However, while this model is very useful, it does not specifically elucidate the pathways between migration and cognition.

### Push and pull theory

The push-pull theory has been widely used in geography and economics research to examine factors that influence people’s decision to migrate [21, 22]. To illustrate, this theory emphasizes the interplay between sending- and receiving place factors that govern the migration process. Push factors often include dissatisfying conditions (e.g. political instability, heavy taxation) in the sending places that motivate people to migrate. In contrast to push factors, pull factors are favorable conditions (e.g. less polluted environment, health care system) in receiving countries that facilitate the migration process. Although the push-pull theory emphasizes that factors in both sending- and receiving places are important to the migration decision, whether these factors can cause accumulation of disease risks and whether migration is associated with certain health outcomes are only vaguely implied in this theory.

In sum, only using one theory is not sufficient to help us elucidate the associations between migration and cognitive function. Therefore, we further reviewed current empirical findings related to migration, cognition, and factors associated with cognitive function, incorporated these empirical findings into the three theories, and ultimately developed a general conceptual framework of the relationships between migration and cognitive function. We did not include all the literature but highlighted several studies that illustrate each potential mechanism.

## Potential mechanisms impacting relationships between migration and cognitive function

### Migration status

An individual’s migration status consists of several intercorrelated key elements, including geographic patterns, age at migration/length of stay, and reasons for migration. A major trend in migration in the world today is that people tend to move from less developed areas (e.g. rural settings, low- and middle-income countries) to more developed places (e.g. urban areas, high-income countries) for more working opportunities, better education, or higher payment [1]. For example, in China, millions of people have migrated from rural areas into cities for employment since the economic reform in 1979 [23]. Similar patterns have also been observed in the immigrant populations: a significant number of laborers from Mexico or other Latin American countries moved to the U.S. and worked in the manufacturing or service sectors [24, 25]. In addition to this type of migration that is often described as upward social mobility, other types of geographic movements also exist, including involuntary migration due to natural disasters, or migration for the purpose of marriage or family union [26–28]. For example, rural to rural migration is commonly seen among women in India; and a significant proportion of this rural to rural migration is related to marriage [29, 30].

Age at migration (or length of stay) is another critical component of an individual’s migration status. First, age at migration is likely to be associated with the length of exposure to certain physical and social environments. Additionally, people tend to migrate for specific reasons at different stages of the life course. For example, upward social mobility is more likely to occur during young adulthood [31].

**Fig. 1:**
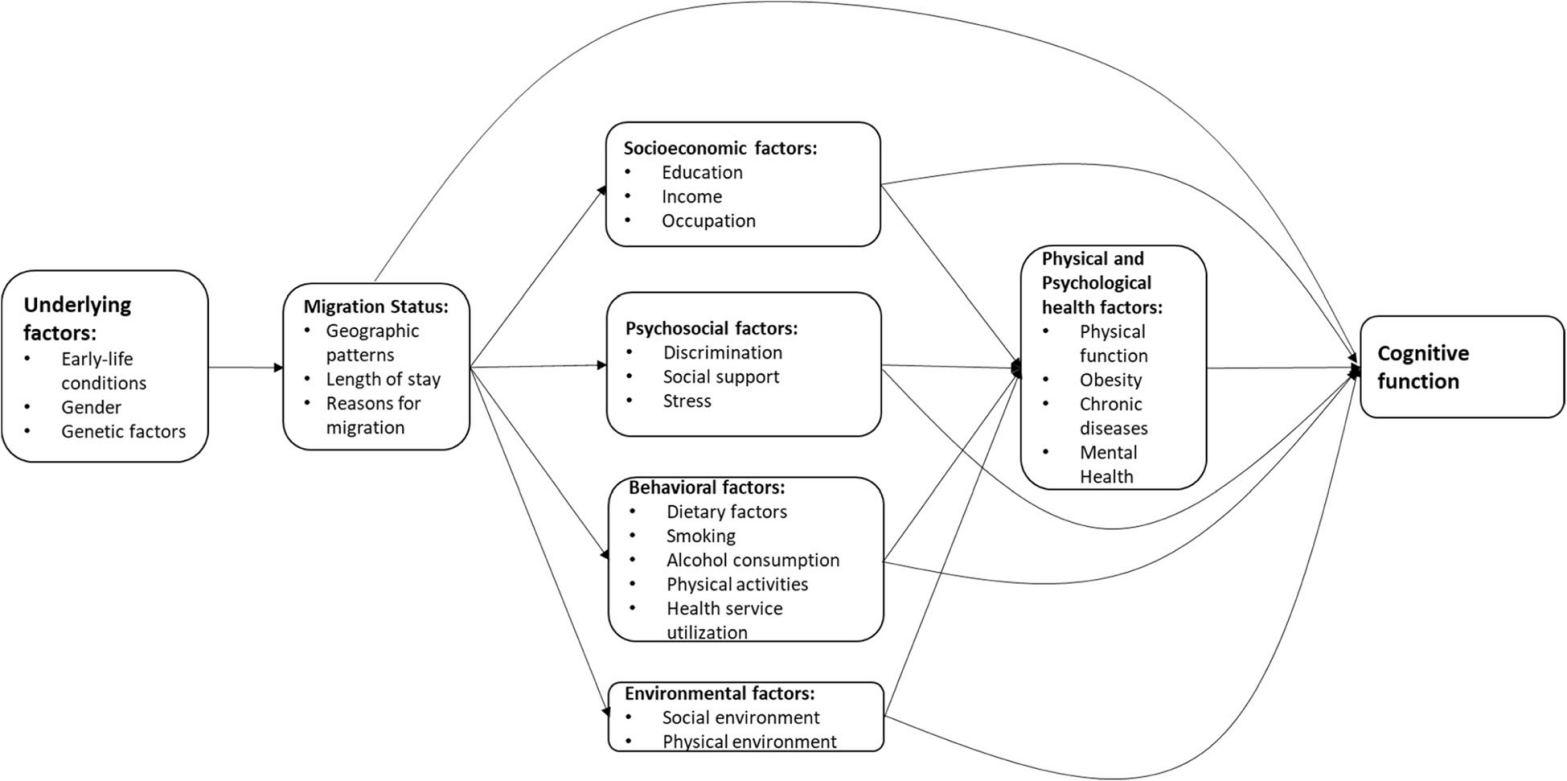
Conceptual Framework of Potential Mechanisms to Explain the Relationships between Migration and Cognitive Function

The potential pathways through which migration affects cognitive function are complex and multifactorial. Different geographic movements and the related reasons for migration are likely to affect cognitive function though different pathways. These potential mechanisms can be categorized in terms of socioeconomic, psychosocial and behavioral factors, physical and psychological health, and environmental factors. Age at migration can determine the duration of exposure that ultimately impacts the magnitude of these effects on cognitive function. Figure 1, which is an extension and elaboration of the figure in our previous systematic review [10], depicts factors that are associated with an individual’s cognitive function and how the migration process may change some of those factors.

### Socioeconomic status (SES)

Socioeconomic factors are often assessed in the literature using measures such as educational attainment, income level, and occupational status [4]. A number of studies have shown that adult SES such as education, income, and occupation are protective factors against cognitive decline [8, 32–34]. Migrants who moved from poorer areas to more developed places are likely to achieve socioeconomic advancement, which is often described as upward social mobility [31].

#### Education

Moving from rural areas into cities, or from developing countries to developed countries, is likely to lead to more or better education opportunities [23, 30]. People who receive higher levels of education demonstrated better cognitive function [35, 36]. One possible explanation is that brain function is stimulated through learning activities or social engagement [37]. Therefore, higher education is related to more cognitive reserve that helps people maintain their brain function [38].

#### Income

In addition to education, research has shown that migrants are likely to receive higher income from their new job after migration than what they could earn back home [1, 23, 39]. Prior research has found that controlling for education and other factors, higher income has been independently associated with higher scores in cognition tests [8, 32]. Higher income level may allow people to afford a good quality diet and better living environment that, in turn, may have a positive impact on cognitive health [40, 41]. It is also possible that migration can lead to an improvement in financial status that increases the likelihood for migrants to obtain health insurance and use healthcare services [42, 43], resulting in more preventative care to reduc the risk of diseases that negatively affect cognitive function in later life [44].

#### Occupation

It is likely that migration results in changes in occupations [23, 30]. Studies have reported the association between a highly-skilled occupation and better cognitive function while adjusting for education and income factors [33, 34]. Occupation often reflects different work exposures and activities [45]. Therefore, migrants may benefit from intellectual stimulation by working on different tasks and learning new skills, which are associated with better cognitive function [35–37, 46].

### Psychosocial factors

Migration involves many psychosocial changes [47]. The following section describes how these migration-related psychosocial changes are associated with cognitive function.

#### Perceived discrimination

A number of studies have documented the hostility and discrimination that migrants experience [48, 49]. For example, rural-to-urban migrants in China have often been denied access to many of the social welfare programs such as health insurance and unemployment benefits that are available to their urban counterparts, even if they were doing the same job [50]. Among immigrants, discrimination and segregation in host countries are also often reported [51, 52]. Perceived discrimination may result in social isolation; and previous studies found that social isolation is a risk factor for cognitive impairment and dementia [53, 54].

#### Social support

Previous research has reported that migrants often experienced various stressful life events, such as separating from family, both during and after the migration process [55]. Family separation is likely related to reduced social support [52, 56, 57]. Small social networks and less social support have been shown to be risk factors forcognitive decline [58–60]. It is also possible that adequate social support and a large social network can facilitate an individual’s access to health care and promote healthy behaviors, ultimately reducing the impact of other risk factors that affect cognitive function [61].

#### Stress

In addition to experiencing reduced social networks and social support, migrants are often under great stress during the migration process [62, 63]. Studies have shown that stressful life events may affect elderly participants' inhibitory control in attentional and sensorimotor domains and therefore influence their cognitive function [64]. However, a longer stay in a hosting place has been shown to be associated with less stress and an improved social network [65]. As a result, the effects of these negative psychosocial factors such as reduced social network and increased stress on cognitive function may decrease as migrants stay longer in hosting places.

### Behavioral factors

Changes in health behaviors are often observed in migrant populations. Migrants from less developed areas are likely to adopt westernized life styles that can negatively affect health, such as high calorie intake, physical inactivity, sedentary employment, and tobacco use [66–68]. These high-risk lifestyles lead to disorders that directly affect an individual’s cognitive function [69, 70], and also serve as mediators in the relationship between migration and cognitive function.

#### Dietary factors

Dietary acculturation has been observed among immigrants [67, 71]. Studies that examined the change of dietary patterns among immigrants in the United States indicate that immigrants tend to consume more calorie-dense food but less fruits and vegetables after arriving in the United States [67, 71]. In addition, a longer stay in the United States has been associated with more westernized dietary patterns [71]. Research shows that similar patterns occur in the rural-to-urban migrant populations in developing countries [72–74]. Previous studies have reported that regular intake of fruits, vegetables, and fiber is associated with better cognitive function [70, 75], while westernized food consumption is related to poorer cognitive performance [76, 77]. Therefore, migration may trigger changes in migrants’ dietary behaviors that generate negative effects on their later-life cognitive function.

#### Smoking

An increasing prevalence of smoking has been observed among migrant populations, which may partially explain the poorer cognitive function found in migrants than those who did not move. Studies in China and Guatemala have indicated that moving into cities is associated with higher likelihood of smoking [78, 79]. Female immigrants appear more likely to smoke than their counterparts who remain in home countries [66, 80]. A wealth of data has shown the negative effect of smoking on cognitive function [81–84], which may occur due to increased risk of cardiovascular diseases and inflammation [85].

#### Alcohol consumption

Unlike smoking, light-to-moderate alcohol consumption has been shown to reduce the risk of developing dementia [86, 87]. One possible explanation is that light-to-moderate drinking might be cardio protective [86, 88]. However, previous research has yielded inconsistent results on the association between heavy drinking and cognitive function [85, 89]. Therefore, the relationship between alcohol consumption and cognitive function might be an inverted U-shape [90]. Still, an increasing trend of alcohol consumption has occurred in both immigrant and internal migrant populations [78, 91].

#### Physical activities

A growing body of literature has demonstrated lower physical activity among rural-to-urban migrants compared to rural residents, which may be explained by sedentary employment in the cities [78, 92]. Similarly, immigrants are also found to be less likely to participate in physical activities, and longer stays in a hosting country will increase this likelihood [93, 94]. Physical activities have long-term positive effects on later-life cognitive function [95–97]. One possible linkage between physical activity and cognition is that physical activity leads to improvements in cardiorespiratory fitness that are beneficial for cognitive function [96].

#### Health services utilization

Despite the negative health behaviors that are related to migration, people who move to more developed areas or countries may improve their access to better healthcare services. It is believed that healthcare in developed countries is generally better than in developing countries [98]. Similarly, in developing countries where massive internal migration occurs, the best healthcare is centralized in urban areas [99, 100]. Although migrants from developing countries or rural areas may have better access to healthcare after migration, the utilization of these services may not be improved immediately. It may take some time for these migrants to be fully aware of and gain access to the available healthcare resources [101, 102].

### Physical and psychological health

As discussed in previous sections, migration may trigger changes in several risk and protective factors, including SES, psychosocial, and behavioral factors. These migration-related factors not only directly interact with cognitive function, but also have impacts on individuals’ physical and psychological health and ultimately influence cognitive function [69, 103].

#### Physical function

A growing body of literature has demonstrated the association between physical function and cognitive function. For example, a recent study has demonstrated that decline in gait and balance function preceded decline in neurological processing speed tasks [104]. Therefore, factors that are associated with an individual’s physical function may have indirect effects on cognitive function. Physical function is associated with several factors such as SES [105, 106], health behaviors [106, 107], and social support [108]. These factors are likely to change during the migration process. Therefore, migration may indirectly affect an individual’s cognitive function through the pathways that we described above.

#### Obesity

Changes in health behaviors, such as adopting a westernized diet and being physical inactive, are likely to increase the risk of obesity in the migrant populations [109, 110]. In addition, a longer stay in the hosting place has been shown to be significantly associated with obesity or overweight [111, 112]. The linkage between obesity and cognitive function is well-established [77, 113]. Therefore, obesity could mediate the effect of health behavior changes that occurred along with the migration process on later-life cognitive function.

#### Chronic diseases

Similar mediating effects can be found in chronic diseases. On one hand, the changes in migration-related health behaviors not only contribute to the risk of obesity and overweight in the migrant populations; these high-risk lifestyles are also associated with the development of chronic diseases like cardiovascular disease and diabetes [83, 114, 115]. On the other hand, if people experience improvement in their SES that is due to migration, they may be more likely to use preventive care [116]. Also, migration can lead to improvement in access to healthcare [99, 100], which can promote better prevention and management of chronic diseases [117]. Increasing evidence suggests that chronic diseases such as hypertension, diabetes, and arrhythmias are related to greater risk of cognitive impairment and dementia [69, 103, 118–122]. Although the precise mechanisms underlying the association between chronic diseases and cognitive function remain unclear, one common explanation is that both micro- and macro-vascular complications increase the risk of cognitive impairment [123].

#### Mental health

Previous research has established linkages between migration-related psychosocial factors and mental health [48, 56, 124, 125]. For example, perceived discrimination has been found to be a risk factor for depressive symptoms [125, 126]. The reduced social networks among immigrants also has been shown to have negative effects on mental health [65, 127]. However, as migrants stay longer in the hosting places, it is possible that they will rebuild their social networks, which will reduce the negative effects on mental health [65]. Additionally, longer stays have been shown to be related to more use of mental health services that would help improve mental health status [128, 129]. Evidence from previous studies suggests that depressive symptoms are associated with mild cognitive impairment and dementia [130, 131]. It is possible that the changes in psychosocial factors during or after migration will negatively impact migrants’ mental health status, and therefore increase the risk of cognitive impairment. However, these negative impacts on cognitive function could be reduced gradually as their length of stay increases.

### Environmental factors

Moving from one area to another often leads to changes in both physical and social environment. The following section discusses the possible linkages between migration, changes in environmental factors, and cognition.

#### Social environment

For immigrants, the similarities in social environment between sending and receiving countries may determine the amount of change that immigrants experience in psychosocial and behavioral factors that are associated with physical and psychosocial health, and, thus, with cognitive function [132]. For example, immigrants from low-income countries are more likely to experience discrimination when they migrate to a high-income country [133]. Behavioral changes among immigrants also differ by the countries of origin. For example, the prevalence of smoking varies between Asian and Latino immigrants in the United States; and the gender gap in smoking prevalence is greater among Asian than Latino immigrants [66]. In addition, country of origin has been shown to be a significant factor that influences changes in dietary patterns and risks of chronic diseases among immigrants [134].

#### Physical environment

Increasing evidence has suggested that certain physical environments can be a potential risk factor for cognitive impairment. Prior studies have found that people who work in agricultural settings are more likely to be exposed to pesticide that increases the risk of cognitive decline [135]. In addition, people who live in rural areas especially in developing countries still often use open fires for cooking [136–138]. Open fire as a major source of indoor pollution has been shown to be associated with poorer cognitive function [139]. Therefore, when people move out of rural areas with these types of environmental exposures, they may protect their cognitive function in later life. However, there is some new evidence suggesting that exposure to air pollution such as particulate matter or traffic-related air pollution in urban areas may accelerate cognitive decline in older ages [140, 141]. As a result, people who move into cities may also face new environmental risk factors for cognitive decline. Research in this area is still in its infancy. Future research should investigate whether migration and cognition could be linked through changes in physical environment.

### Underlying Factors

In addition to many factors that may change during the migration process, there are other time-invariant factors that may affect an individual’s cognitive function. For example, early-life conditions, gender, and genetic factors may each affect cognitive function via various pathways.

#### Early-life conditions

Studies have shown that early-life exposures to negative events (e.g. hunger and malnutrition) are likely to increase the risk of cognitive decline [142, 143]. People who live in low- and middle-income countries are more likely to experience negative early-life exposures. Even people who survived such negative exposures (e.g. infectious diseases) during their childhood, show a higher risk for developing cognitive impairment later on than those who didn’t experience negative exposures [144]. In addition, a parent’s educational level has been shown to influence the trajectories of cognitive aging [145, 146]. Recent studies demonstrated that physical measures in early life such as birth length and head circumference are also associated with later-life cognitive function [142, 147]. It is possible that these measures indicate early brain development, which accounts for nearly 50% of a person’s total cognitive ability [148].

#### Gender

Prior research has found that women reported worse cognitive function than men, especially in the oldest old age range [149, 150]. Gender is an underlying factor that influences the relationship between migration and cognitive function through several pathways. First, the migration patterns may differ between men and women. For example, in India, gender differences have been observed in the migration trends. In male populations, rural-to-urban migration is the largest stream (39.0%) and employment and education are the two main reasons for this type of migration [30]. However, in women, rural-to-rural migration is the predominant stream accounting for 70% of all the female migration, primarily for the purpose of marriage [29, 30]. Therefore, changes in SES between male and female migrants may be different due the reasons for migration and geographic patterns: in developing countries, men are more likely to experience upward social mobility [28], and ultimately have better cognitive function [149, 150].

Gender differences have also been found in changes in health behaviors. For example, research indicates that male immigrants are more likely to adapted to westernized diets than their female counterparts [67, 71]. Additionally, the impact of migration on an individual’s smoking behavior differs by gender, with male migrants less likely to smoke than females [66, 80]. As a result, the gender differences in health behaviors among migrants may produce different influences on later-life cognitive function.

#### Genetic factors

Research has established linkages between dementia and two genes—apoliporotein E (APOE) and neuronal sortilin-related receptor (SORL1) [151–153]. Studies have shown that APOE ε4 increases the risk of dementia. However, the effect varies by sex, race/ethnicity, age, and geographic location [151, 154–157]. The SORL1 gene has been found as the second most important gene related to cognitive function [151, 158]. Studies that covered a broad range of locations and ethnicity groups have shown SORL1 is a risk gene in cognitive decline [151, 159, 160].

## Conclusions

The association between migration and cognition is multidimensional and complex. This study identifies several pathways which potentially explain the linkages between migration and later-life cognitive function. An individual’s cognitive function is associated with SES, psychosocial and behavioral factors, and physical and psychological health status. These factors mediate the relationship between migration and cognitive function. The migration process may lead to changes in SES, psychosocial and behavioral factors, and these changes will either positively or negatively influence an individual’s cognitive function. Such changes may also impact cognitive function indirectly by improving or harming an individual’s physical and psychological health. Age at migration (or the length of stay in the new location) is associated with levels of change in SES, psychosocial and behavioral factors, and physical and psychological health status. Additionally, environmental factors may potentially mediate the relationship between migration and cognition. Underlying factors, such as an individual’s early-life exposures, gender, or genetic factors, which will not change through migration, are also related to later-life cognitive function.

This conceptual framework has potential implications for clinical practice and global health policies. First, finding from this paper supports the trends towards ‘needs based’ rather than age-determined health and social services in countries with both large aging population and migrant populations. Both primary health care providers and policy makers should be aware that cognitive impairment/decline may be more commonly experienced in certain migrant populations. Therefore, it is crucial to promote early screening for potential cognitive impairment in clinical practice and making sure this practice covers these migrant populations.

In addition, factors that are identified in this framework that can potentially be used to design tailored interventions or programs to promote cognitive health. For example, migration can be a stressful event that may have negative impacts on individuals’ psychological well-being. In this case, migrants might benefit from interventions such as community-based psychological services that help them cope with stress and improve mood. In addition to that, place-based social activities might be helpful to some migrants to expand their social networks, which might have positive impacts on their cognitive function. We also identified gender to be a potential underlying factor. This finding highlights the needs of designing and implementing programs to promote gender equality and to empower all women and girls in multiple aspects (the Sustainable Development Goal 5). Overall, one of the main outcomes of this paper is a conceptual framework of the potential mechanisms linking migration and cognitive function and related underlying factors. Using this framework, the relative importance of the identified pathways may be empirically refined, tested, and validated.

## Abbreviations

APOE: Apoliporotein E
SES: Socioeconomic status
SORL1: Neuronal sortilin-related receptor

## References

[1] International Organization for Migration (2015). World Migration Report.

[2] United Nations Department of Economic and Social Affairs (2013). Global Migration: Demographic Aspects and Its Relevance for Development.

[3] World Health Organization. World report on ageing and health. Geneva: World Heal. Organ; 2015.

[4] Treas J, Gubernskaya Z. Immigration, Aging, and the Life Course. In: Handbook of Aging and the Social Sciences. Academic Press; 2016. p. 143–61. 10.1016/B978-0-12-417235-7.00007-X.

[5] Spallek J, Zeeb H, Razum O, Schenker MB, Castañeda X, Rodriguez-Lainz A (2014). Life Course Epidemiology A Conceptual Model for the Study of Migration and Health. Migr. Heal. A reserach Methods Handb.

[6] Prince M, Wimo A, Guerchet M, Gemma-Claire A, Wu Y-T, Prina M. World Alzheimer Report 2015: The Global Impact of Dementia - An analysis of prevalence, incidence, cost and trends: Alzheimer’s Dis Int; 2015. p. 84. London.

[7] Prince M, Comas-Herrera MA, Knapp M, Guerchet M, Karagiannidou MM (2016). World Alzheimer Report 2016 Improving healthcare for people living with dementia coverage, QualIty and costs now and In the future.

[8] Deary IJ, Corley J, Gow AJ, Harris SE, Houlihan LM, Marioni RE (2009). Age-associated cognitive decline. Br. Med. Bull.

[9] Livingston G, Sommerlad A, Orgeta V, Costafreda SG, Huntley J, Ames D, et al. Dementia prevention, intervention, and care. Lancet (London, England) [Internet]. Elsevier; 2017 [cited 2017 Jul 20];0. Available from: http://www.ncbi.nlm.nih.gov/pubmed/28735855 10.1016/S0140-6736(17)31363-6 28735855

[10] Xu H, Zhang Y, Wu B. Association between migration and cognitive status among middle-aged and older adults: a systematic review. BMC Geriatr. 2017; [Internet] [cited 2017 Aug 22];17:184. Available from: http://bmcgeriatr.biomedcentral.com/articles/10.1186/s12877-017-0585-2. 10.1186/s12877-017-0585-2 PMC5561610 28818064

[11] Glick-Schiller N, Basch L, Szanton BC. From Immigrant to Transmigrant: Theorizing Transnational Migration. Anthropol. Q. 1995;68:48–63 [Internet]. Available from: https://www.jstor.org/stable/pdf/3317464.pdf.

[12] Schiller NG (2005). Transnational social fields and imperialism: Bringing a theory of power to Transnational Studies. Anthropol Theory.

[13] Orth U, Robins R, Widaman K. Life-span development of self-esteem and its effects on important life outcomes. J Pers Soc Psychol. 2012;102:1271–88. 10.1037/a0025558 21942279

[14] Liu G, Dupre ME. Health Trajectories of Women in China: The Role of Parental Caregiving. J Gerontol B Psychol Sci Soc Sci. 2014:1–12 [Internet]. Available from: http://www.ncbi.nlm.nih.gov/pubmed/25315160. 10.1093/geronb/gbu144 25315160

[15] Elder GH Jr., Johnson MK, Crosnoe R. The Emergence and Development of the Life Course. In Handbook of the Life Course, eds. J. Mortimer and M. J. Shanahan (H.Kaplan, series, editor) New York: Plenum Publishing; 2003.

[16] Kuh D, Ben-Shlomo Y, Lynch J, Hallqvist J, Power C (2003). Life course epidemiology. J Epidemiol Commun Health.

[17] Wilkinson R, Marmot M (2003). Determinants of Health the Solid Facts. World Health.

[18] Marmot M (2005). Public Health Social determinants of health inequalities. Lancet.

[19] Havranek EP, Mujahid MS, Barr DA, Blair IV, Cohen MS, Cruz-Flores S, et al. Social Determinants of Risk and Outcomes for Cardiovascular Disease: A Scientific Statement From the American Heart Association. Circulation. 2015; [Internet] [cited 2015 Oct 19];132:873–98. Available from: http://www.ncbi.nlm.nih.gov/pubmed/26240271. 10.1161/CIR.0000000000000228 26240271

[20] Dyck RF, Karunanayake C, Janzen B, Lawson J, Ramsden VR, Rennie DC, et al. Do discrimination, residential school attendance and cultural disruption add to individual-level diabetes risk among Aboriginal people in Canada? BMC Public Health. 2015;15:1222. 10.1186/s12889-015-2551-2. 10.1186/s12889-015-2551-2 PMC4675031 26651995

[21] Dorigo G, Tobler W (1983). Push-Pull Migration Laws. Ann Assoc Am.

[22] Ravenstein EG (1885). The laws of migration. J Stat Soc London.

[23] Chan KW. Internal labor migration in China: Trends, geography and policies. Popul Distrib Urban Intern Migr Dev An Int Perspect. 2012:81–102.

[24] Chavez Juarez FW (2009). Determinants of internal migration in Mexico at an aggregated and a disaggregated level.

[25] Garduño R, Baylis K, PArends-KUENNING M. Regional Economic Analysis of Internal Migration in Mexico. 2011; Available from: https://old.reunionesdeestudiosregionales.org/Oviedo2013/htdocs/pdf/p649.pdf.

[26] Myers CA, Slack T, Singelmann J. Social vulnerability and migration in the wake of disaster: the case of Hurricanes Katrina and Rita. Popul. Environ: Springer Netherlands; 2008. [Internet]. [cited 2016 Jul 13];29:271–91. Available from: http://link.springer.com/10.1007/s11111-008-0072-y

[27] Pedraza S (1991). Women and Migration : The Social Consequences of Gender. Annu Rev Sociol.

[28] Tienda M, Booth K. Gender, Migration and Social Change Int Sociol. SAGE Publications; 1991 [Internet]. [cited 2016 Jul 13];6:51–72. Available from: http://iss.sagepub.com/cgi/doi/10.1177/026858091006001004 10.1177/026858091006001004 12179889

[29] Mohapatra S. Almost a third of Indians, or over 300 million people, are migrants. World Bank. 2010 [Internet] [cited 2015 Dec 19]. Available from: http://blogs.worldbank.org/peoplemove/almost-a-third-of-indians-or-over-300-million-people-are-migrants

[30] Bhagat RB. Urban Migration Trends, Challenges and Opportunities in India. World Migr. Rep. 2015 Migrants Cities New Partnerships to Manag. Mobil. Geveva; 2015.

[31] Alcántara C, Chen C-N, Alegría M (2014). Do post-migration perceptions of social mobility matter for Latino immigrant health?. Soc Sci Med.

[32] Koster A, Penninx BWJH, Bosma H, Kempen GIJM, Newman AB, Rubin SM (2005). Socioeconomic differences in cognitive decline and the role of biomedical factors. Ann. Epidemiol.

[33] Potter GG, Plassman BL, Helms MJ, Foster SM, Edwards NW (2006). Occupational characteristics and cognitive performance among elderly male twins. Neurology.

[34] Gow AJ, Avlund K, Mortensen EL (2014). Occupational characteristics and cognitive aging in the Glostrup 1914 Cohort. J Gerontol Ser B Psychol Sci Soc Sci.

[35] Cagney KA, Lauderdale DS. Education, wealth, and cognitive function in later life. J. Gerontol. B. Psychol. Sci. Soc. Sci. [Internet]. 2002;57:P163-72. Available from: http://www.ncbi.nlm.nih.gov/pubmed/11867664 10.1093/geronb/57.2.p163 11867664

[36] Lee S, Buring JE, Cook NR, Grodstein F (2006). The relation of education and income to cognitive function among professional women. Neuroepidemiology.

[37] Fratiglioni L, Paillard-Borg S, Winblad B (2004). An active and socially integrated lifestyle in late life might protect against dementia. Lancet Neurol.

[38] Valenzuela MJ, Sachdev P (2006). Brain reserve and dementia: A systematic review. Psychol Med.

[39] Garcia AJ, Pindolia DK, Lopiano KK, Tatem AJ (2015). Modeling internal migration flows in sub-Saharan Africa using census microdata. Migr. Stud.

[40] Golant SM. Low-income elderly homeowners in very old dwellings: the need for public policy debate. J Aging Soc Policy. 2008; [Internet]. [cited 2018 Mar 4];20:1–28. Available from: http://www.ncbi.nlm.nih.gov/pubmed/18198157. 10.1300/j031v20n01_01 18198157

[41] Shatenstein B, Ferland G, Belleville S, Gray-Donald K, Kergoat M-J, Morais J, et al. Diet quality and cognition among older adults from the NuAge study. Exp Gerontol Pergamon. 2012; [Internet]. [cited 2018 Mar 4];47:353–60. Available from: https://www.sciencedirect.com/science/article/pii/S0531556512000332#bbb0075. 10.1016/j.exger.2012.02.002 22386581

[42] Chen J. Chronic conditions and receipt of treatment among urbanized rural residents in China. Biomed Res Int. 2013;2013. 10.1155/2013/568959 PMC3753763 23998126

[43] Richter M, Chersich M, Vearey J, Sartorius B, Temmerman M, Luchters S. Migration Status, Work Conditions and Health Utilization of Female Sex Workers in Three South African Cities. J Immigr Minor Heal. [Internet]. 2014;16:7–17. Available from: 10.1007/s10903-012-9758-4%5Cnhttp://search.ebscohost.com/login.aspx?direct=true&db=a9h&AN=93750799&lang=es&site=ehost-live. 10.1007/s10903-012-9758-4 PMC3895178 23238581

[44] Pottie K, Rahal R, Jaramillo A, Birtwhistle R, Thombs BD, Singh H, et al. Recommendations on screening for cognitive impairment in older adults. CMAJ. 2016; [cited 2016 Jul 11];188:37–46. [Internet] Available from: http://www.ncbi.nlm.nih.gov/pubmed/26622001. 10.1503/cmaj.141165 PMC4695353 26622001

[45] Winkleby MA, Jatulis DE, Frank E, Fortmann SP. Socioeconomic status and health: how education, income, and occupation contribute to risk factors for cardiovascular disease. Am. J. Public Health [Internet]. American Public Health Association; 1992 [cited 2018 Mar 4];82:816–20. Available from: http://www.ncbi.nlm.nih.gov/pubmed/1585961 10.2105/ajph.82.6.816 PMC1694190 1585961

[46] Schooler C, Mulatu MS, Oates G (2004). Occupational Self-Direction, Intellectual Functioning, and Self-Directed Orientation in Older Workers: Findings and Implications for Individuals and Societies. Am J Sociol.

[47] Bhugra D. Migration, distress and cultural identity. Br. Med. Bull. [Internet]. Oxford University Press; 2004 [cited 2016 Jul 13];69:129–41. Available from: https://www.ncbi.nlm.nih.gov/pubmed/15226202 10.1093/bmb/ldh007 15226202

[48] Chou K-L (2012). Perceived discrimination and depression among new migrants to Hong Kong: The moderating role of social support and neighborhood collective efficacy. J Affect Disord.

[49] Zhong BL, Liu TB, Huang JX, Fung HH, Chan SSM, Conwell Y, et al. Acculturative stress of Chinese rural-to-urban migrant workers: A qualitative study. Laks J, editor. PLoS One [Internet]. Public Library of Science; 2016;11:e0157530. [cited 2016 Jul 13] Available from: http://dx.plos.org/10.1371/journal.pone.0157530 10.1371/journal.pone.0157530 PMC4907425 27300005

[50] Meng X, Manning C, Li S, Effendi TN (2010). The Great Migration: Rural–Urban Migration in China and Indonesia.

[51] Flippen CA, Parrado EA. Perceived discrimination among Latino immigrants in new destinations: The case of Durham, NC. Sociol. Perspect. [Internet]. NIH Public Access; 2015;58:666–85 [cited 2016 Jul 13]. Available from: http://www.ncbi.nlm.nih.gov/pubmed/26848208 10.1177/0731121415574397 PMC4734395 26848208

[52] Negi NJ. Battling Discrimination and Social Isolation: Psychological Distress Among Latino Day Laborers. Am. J. Community Psychol. [Internet]. Springer US; 2013;51:164–74 [cited 2016 Jul 13]. Available from: http://doi.wiley.com/10.1007/s10464-012-9548-0 10.1007/s10464-012-9548-0 22864958

[53] DiNapoli EA, Wu B, Scogin F. Social Isolation and Cognitive Function in Appalachian Older Adults. Res Aging 2014;36:161–179. [Internet] Available from: http://roa.sagepub.com/cgi/doi/10.1177/0164027512470704 10.1177/0164027512470704 25650688

[54] Shankar A, Hamer M. Social isolation and loneliness: relationships with cognitive function during 4 years of follow-up in the English Longitudinal Study of Ageing. Psychosom Med. 2013 75:161–70 [Internet] [cited 2015 Dec 20]. Available from: http://journals.lww.com/psychosomaticmedicine/Abstract/2013/02000/Social_Isolation_and_Loneliness___Relationships.9.aspx 10.1097/PSY.0b013e31827f09cd 23362501

[55] Lu Y, Hu P, Treiman DJ (2012). Migration and depressive symptoms in migrant-sending areas: Findings from the survey of internal migration and health in China. Int J Public Health.

[56] Jasinskaja-Lahti I (2006). Perceived Discrimination, Social Support Networks, and Psychological Well-being Among Three Immigrant Groups. J Cross Cult Psychol.

[57] Hurtado-de-Mendoza A, Gonzales FA, Serrano A, Kaltman S. Social Isolation and Perceived Barriers to Establishing Social Networks Among Latina Immigrants. Am. J. Community Psychol. [Internet]. Springer US; 2014 53:73–82 [cited 2016 Jul 13]. Available from: http://doi.wiley.com/10.1007/s10464-013-9619-x 10.1007/s10464-013-9619-x 24402726

[58] Crooks VC, Lubben J, Petitti DB, Little D, Chiu V (2008). Social network, cognitive function, and dementia incidence among elderly women. Am J Public Health.

[59] Yeh S-CJ, Liu Y-Y (2003). Influence of social support on cognitive function in the elderly. BMC Health Serv Res.

[60] Zhu S, Hu J, Efird JT (2012). Role of social support in cognitive function among elders. J Clin Nurs.

[61] Kuiper J, Zuidersma M, Oude Voshaar R, Zuidema S, van den Heuvel E, Stolk R (2015). Social relationships and risk of dementia: A systematic review and meta-analysis of longitudinal cohort studies. Ageing Res Rev.

[62] Aroian KJ, Norris AE. Resilience, Stress, and Depression among Russian Immigrants to Israel. West. J. Nurs. Res. [Internet]. SAGE Publications; 2000 22:54–67 [cited 2016 Jul 13]. Available from: http://wjn.sagepub.com/cgi/doi/10.1177/01939450022044269

[63] Hattar-Pollara M, Meleis AI. The Stress of Immigration and the Daily Lived Experiences of Jordanian Immigrant Women in the United States. West. J. Nurs. Res. [Internet]. SAGE Publications; 1995 17:521–39 [cited 2016 Jul 13]. Available from: http://wjn.sagepub.com/cgi/doi/10.1177/019394599501700505 10.1177/019394599501700505 7571553

[64] Marshall AC, Cooper NR, Geeraert N (2015). Experienced Stress Produces Inhibitory Deficits in Elderlies’ Flanker Task Performance: First Evidence for Lifetime Stress Effects Beyond Memory. Biol Psychol.

[65] Kim BJ, Sangalang CC, Kihl T (2012). Effects of acculturation and social network support on depression among elderly Korean immigrants. Aging Ment Health Routledge.

[66] Gorman BK, Lariscy JT, Kaushik C (2014). Gender, acculturation, and smoking behavior among U.S. Asian and Latino immigrants. Soc Sci Med.

[67] Akresh IR. Dietary assimilation and health among hispanic immigrants to the United States. J. Health Soc. Behav. [Internet]. SAGE Publications; 2007 [cited 2016 Jul 12];48:404–17. Available from: http://www.ncbi.nlm.nih.gov/pubmed/18198687 10.1177/002214650704800405 18198687

[68] Allen JD, Caspi C, Yang M, Leyva B, Stoddard AM, Tamers S (2014). Pathways between acculturation and health behaviors among residents of low-income housing: The mediating role of social and contextual factors. Soc Sci Med.

[69] Tuligenga RH, Dugravot A, Tabák AG, Elbaz A, Brunner EJ, Kivimäki M (2014). Midlife type 2 diabetes and poor glycaemic control as risk factors for cognitive decline in early old age: A post-hoc analysis of the Whitehall II cohort study. Lancet Diabetes Endocrinol.

[70] Otaegui-Arrazola A, Amiano P, Elbusto A, Urdaneta E, Martínez-Lage P (2014). Diet, cognition, and Alzheimer’s disease: Food for thought. Eur J Nutr.

[71] Cuy Castellanos D. Dietary Acculturation in Latinos/Hispanics in the United States. Am. J. Lifestyle Med. [Internet]. SAGE Publications; 2015 [cited 2016 Jul 12];9:31–6. Available from: http://ajl.sagepub.com/cgi/doi/10.1177/1559827614552960

[72] Skoro R, Smith C, Jin Y, Feng Q (2014). Foodways and the Floating Population: Diet and Rural-to-Urban Migration in Nanjing, China. J Nutr Ecol Food Res American Scientific Publishers.

[73] Unwin NN, James P, McLarty DD, Machybia H, Nkulila P, Tamin B (2010). Rural to urban migration and changes in cardiovascular risk factors in Tanzania: a prospective cohort study. BMC Public Health BioMed Central.

[74] Bowen L, Ebrahim S, De Stavola B, Ness A, Kinra S, Bharathi AV (2011). Dietary Intake and Rural-Urban Migration in India: A Cross-Sectional Study. Earnest CP, editor. PLoS One.

[75] Luchsinger JA, Noble JM, Scarmeas N (2007). Diet and Alzheimer’s disease. Curr Neurol Neurosci Rep.

[76] Torres SJ, Lautenschlager NT, Wattanapenpaiboon N, Greenop KR, Beer C, Flicker L, et al. Dietary patterns are associated with cognition among older people with mild cognitive impairment. Nutrients [Internet]. Multidisciplinary Digital Publishing Institute (MDPI); 2012 [cited 2016 Jul 12];4:1542–51. Available from: http://www.ncbi.nlm.nih.gov/pubmed/23201831 10.3390/nu4111542 PMC3509504 23201831

[77] Kanoski SE, Davidson TL. Western diet consumption and cognitive impairment: links to hippocampal dysfunction and obesity. Physiol. Behav. [Internet]. NIH Public Access; 2011 [cited 2016 Jul 12];103:59–68. Available from: http://www.ncbi.nlm.nih.gov/pubmed/21167850 10.1016/j.physbeh.2010.12.003 PMC3056912 21167850

[78] Torun B, Stein AD, Schroeder D, Grajeda R, Conlisk A, Rodriguez M, et al. Rural-to-urban migration and cardiovascular disease risk factors in young Guatemalan adults. Int. J. Epidemiol. [Internet]. Oxford University Press; 2002 [cited 2015 Nov 16];31:218–26. Available from: http://www.ncbi.nlm.nih.gov/pubmed/11914324 10.1093/ije/31.1.218 11914324

[79] Cui X, Rockett IRH, Yang T, Cao R (2012). Work stress, life stress, and smoking among rural-urban migrant workers in China. BMC Public Health BioMed Central.

[80] Choi S, Rankin S, Stewart A, Oka R (2008). Effects of acculturation on smoking behavior in Asian Americans: a meta-analysis. J Cardiovasc Nurs.

[81] Anstey KJ, Von Sanden C, Salim A, O’Kearney R (2007). Smoking as a risk factor for dementia and cognitive decline: A meta-analysis of prospective studies. Am J Epidemiol.

[82] Peters R, Poulter R, Warner J, Beckett N, Burch L, Bulpitt C (2008). Smoking, dementia and cognitive decline in the elderly, a systematic review. BMC Geriatr.

[83] Sabia S, Elbaz A, Dugravot A, Head J, Shipley M, Hagger-Johnson G (2012). Impact of smoking on cognitive decline in early old age: the Whitehall II cohort study. Arch Gen Psychiatry.

[84] Nooyens ACJ, van Gelder BM, Verschuren WMM (2008). Smoking and cognitive decline among middle-aged men and women: the Doetinchem Cohort Study. Am J Public Health.

[85] Horvat P, Richards M, Kubinova R, Pajak A, Malyutina S, Shishkin S (2015). Alcohol consumption, drinking patterns, and cognitive function in older Eastern European adults. Neurology.

[86] Ronksley PE, Brien SE, Turner BJ, Mukamal KJ, Ghali WA (2011). Association of alcohol consumption with selected cardiovascular disease outcomes: a systematic review and meta-analysis. BMJ.

[87] Peters R, Peters J, Warner J, Beckett N, Bulpitt C (2008). Alcohol, dementia and cognitive decline in the elderly: A systematic review. Age Ageing.

[88] Roerecke M, Rehm J (2012). The cardioprotective association of average alcohol consumption and ischaemic heart disease: A systematic review and meta-analysis. Addiction.

[89] Kesse-Guyot E, Andreeva VA, Jeandel C, Ferry M, Touvier M, Hercberg S, et al. Alcohol consumption in midlife and cognitive performance assessed 13 years later in the SU.VI.MAX 2 cohort. PLoS One. 2012:7–e52311 [Internet] Available from: http://www.pubmedcentral.nih.gov/articlerender.fcgi?artid=3526526&tool=pmcentrez&rendertype=abstract. 10.1371/journal.pone.0052311 PMC3526526 23284983

[90] Horvat P. Life course socioeconomic position, health behaviours and cognitive function in middle-aged and older persons in four Central and Eastern European populations: University College London; 2014. [cited 2015 [Internet] Dec 19]. Available from: http://discovery.ucl.ac.uk/1420271/

[91] Bryant AN, Kim G. The relation between acculturation and alcohol consumption patterns among older Asian and Hispanic immigrants: Taylor & Francis Group; 2013. 10.1080/13607863.2012.727382 10.1080/13607863.2012.727382 23098103

[92] Yamauchi T, Umezaki M (2005). Rural-urban migration and changing physical activity among Papua New Guinea highlanders from the perspective of energy expenditure and time use. Environ Sci.

[93] Tremblay MS, Bryan SN, Pérez CE, Ardern CI, Katzmarzyk PT. Physical activity and immigrant status: evidence from the Canadian Community Health Survey. Can. J. public Heal. = Rev. Can. santé publique [Internet]. 2006 [cited 2016 Jul 12];97:277–82. Available from: http://www.ncbi.nlm.nih.gov/pubmed/16967745 10.1007/BF03405603 PMC6976030 16967745

[94] Dogra S, Meisner BA, Ardern CI, Buron A, Cots F, Garcia O (2010). Variation in mode of physical activity by ethnicity and time since immigration: a cross-sectional analysis. Int J Behav Nutr Phys Act BioMed Central.

[95] Tolppanen A-M, Solomon A, Kulmala J, Kåreholt I, Ngandu T, Rusanen M (2014). Leisure-time physical activity from mid- to late life, body mass index, and risk of dementia. Alzheimers Dement.

[96] Zhu N, Jacobs DR, Schreiner PJ, Yaffe K, Bryan N, Launer LJ (2014). Cardiorespiratory fitness and cognitive function in middle age: the CARDIA study. Neurology.

[97] Angevaren M, Aufdemkampe G, Verhaar HJJ, Aleman A, Vanhees L. Physical activity and enhanced fitness to improve cognitive function in older people without known cognitive impairment. Cochrane database Syst Rev. 2008:CD005381 Available from: http://www.ncbi.nlm.nih.gov/pubmed/18646126. 10.1002/14651858.CD005381.pub2 18425918

[98] Peters DH, Garg A, Bloom G, Walker DG, Brieger WR, Hafizur RM (2008). Poverty and access to health care in developing countries. Ann N Y Acad Sci.

[99] Vlahov D, Freudenberg N, Proietti F, Ompad D, Quinn A, Nandi V, et al. Urban as a Determinant of Health. J. Urban Heal. [Internet]. Springer US; 2007 [cited 2016 Jul 18];84:16–26. Available from: http://link.springer.com/10.1007/s11524-007-9169-3 10.1007/s11524-007-9169-3 PMC1891649 17356903

[100] Zhang X, Kanbur R (2005). Spatial inequality in education and health care in China. China Econ Rev.

[101] Newbold B (2005). Health status and health care of immigrants in Canada: a longitudinal analysis. J Health Serv Res Policy.

[102] Lu Y (2010). Rural-urban migration and health: Evidence from longitudinal data in Indonesia. Soc Sci Med.

[103] Helmes E, Ostbye T, Steenhuis RE. Association between diabetes and cognition in older adults without dementia. J Aging Res Clin Pract SCIRP. 2013; [cited 2015 Dec 28]. Available from: https://researchonline.jcu.edu.au/29356/.

[104] Finkel D, Ernsth-Bravell M, Pedersen NL (2016). Temporal Dynamics of Motor Functioning and Cognitive Aging. J Gerontol A Biol Sci Med Sci.

[105] Beydoun MA, Popkin BM (2005). The impact of socio-economic factors on functional status decline among community-dwelling older adults in China. Soc Sci Med.

[106] Smith KV, Goldman N (2007). Socioeconomic differences in health among older adults in Mexico. Soc Sci Med.

[107] Seeman TE, Merkin SSS, Crimmins EM, Karlamangla AS (2010). Disability trends among older Americans: National Health and Nutrition Examination surveys, 1988-1994 and 1999-2004. Am J Public Health.

[108] Ashida S, Heaney CA (2008). Differential associations of social support and social connectedness with structural features of social networks and the health status of older adults. J Aging Health.

[109] Commodore-Mensah Y, Samuel LJ, Dennison-Himmelfarb CR, Agyemang C (2014). Hypertension and overweight/obesity in Ghanaians and Nigerians living in West Africa and industrialized countries. J. Hypertens.

[110] Ebrahim S, Kinra S, Bowen L, Andersen E, Ben-Shlomo Y, Lyngdoh T (2010). The effect of rural-to-urban migration on obesity and diabetes in India: a cross-sectional study. PLoS Med.

[111] Goel MS, McCarthy EP, Phillips RS, Wee CC (2004). Obesity among US immigrant subgroups by duration of residence. JAMA.

[112] Delavari M, Sønderlund A, Swinburn B, Mellor D, Renzaho A, Finucane M (2013). Acculturation and obesity among migrant populations in high income countries – a systematic review. BMC Public Health BioMed Central.

[113] Nguyen JCD, Killcross AS, Jenkins TA (2014). Obesity and cognitive decline: role of inflammation and vascular changes. Front Neurosci Frontiers Media SA.

[114] Afable-Munsuz A, Mayeda ER, Pérez-Stable EJ, Haan MN (2014). Immigrant generation and diabetes risk among Mexican Americans: the Sacramento area Latino study on aging. Am J Public Health.

[115] Buja A, Gini R, Visca M, Damiani G, Federico B, Francesconi P, et al. Prevalence of chronic diseases by immigrant status and disparities in chronic disease management in immigrants: a population-based cohort study, Valore Project. BMC Public Health. 2013;13:–504 [cited 2015 Nov 16]. Available from: http://www.pubmedcentral.nih.gov/articlerender.fcgi?artid=3673842&tool=pmcentrez&rendertype=abstract. 10.1186/1471-2458-13-504 PMC3673842 23706129

[116] Pylypchuk Y, Hudson J (2009). Immigrants and the use of preventive care in the United States. Health Econ.

[117] Beaglehole R, Epping-Jordan J, Patel V, Chopra M, Ebrahim S, Kidd M (2008). Improving the prevention and management of chronic disease in low-income and middle-income countries: a priority for primary health care. Lancet.

[118] Kaffashian S, Dugravot A, Nabi H, Batty GD, Brunner E, Kivimäki M (2011). Predictive utility of the Framingham general cardiovascular disease risk profile for cognitive function: evidence from the Whitehall II study. Eur Heart J.

[119] Iadecola C. Hypertension and dementia. Hypertension. 2014;64:3–5 [cited 2015 Dec 20];. Available from: https://www.ncbi.nlm.nih.gov/pubmed/24777976. 10.1161/HYPERTENSIONAHA.114.03040 PMC4234111 24777976

[120] Knopman DS, Roberts RO, Geda YE, Boeve BF, Pankratz VS, Cha RH, et al. Association of prior stroke with cognitive function and cognitive impairment: a population-based study. Arch. Neurol. [Internet]. American Medical Association; 2009 [cited 2015 Dec 20];66:614–9. Available from: http://archneur.jamanetwork.com/article.aspx?articleid=797069 10.1001/archneurol.2009.30 PMC3050015 19433661

[121] Ballard C, Rowan E, Stephens S, Kalaria R, Kenny RA (2003). Prospective follow-up study between 3 and 15 months after stroke: improvements and decline in cognitive function among dementia-free stroke survivors >75 years of age. Stroke.

[122] Kaffashian S, Dugravot A, Brunner EJ, Sabia S, Ankri J, Kivimäki M (2013). Midlife stroke risk and cognitive decline: A 10-year follow-up of the Whitehall II cohort study. Alzheimer’s Dement.

[123] Exalto LG, Whitmer RA, Kappele LJ, Biessels GJ (2012). An update on type 2 diabetes, vascular dementia and Alzheimer’s disease. Exp Gerontol.

[124] Leong F, Park YS, Kalibatseva Z (2013). Disentangling immigrant status in mental health: Psychological protective and risk factors among Latino and Asian American immigrants. Am J Orthopsychiatry.

[125] Edge S, Newbold B. Discrimination and the Health of Immigrants and Refugees: Exploring Canada’s Evidence Base and Directions for Future Research in Newcomer Receiving Countries. J. Immigr. Minor. Heal. [Internet]. Springer US; 2013 [cited 2016 Jul 13];15:141–8. Available from: http://link.springer.com/10.1007/s10903-012-9640-4 10.1007/s10903-012-9640-4 22729289

[126] Mewes R, Asbrock F, Laskawi J (2015). Perceived discrimination and impaired mental health in Turkish immigrants and their descendents in Germany. Compr Psychiatry.

[127] Guruge S, Thomson MS, George U, Chaze F (2015). Social support, social conflict, and immigrant women’s mental health in a Canadian context: a scoping review. J Psychiatr Ment Health Nurs.

[128] Park SY, Cho S, Park Y, Bernstein KS, Shin JK. Factors associated with mental health service utilization among Korean American immigrants. Community Ment. Health J. [Internet]. NIH Public Access; 2013 [cited 2016 Jul 18];49:765–73. Available from: http://www.ncbi.nlm.nih.gov/pubmed/23417654 10.1007/s10597-013-9604-8 PMC3976602 23417654

[129] Leu J, Yen IH, Gansky SA, Walton E, Adler NE, Takeuchi DT (2008). The association between subjective social status and mental health among Asian immigrants: Investigating the influence of age at immigration. Soc Sci Med.

[130] Jorm AF. History of depression as a risk factor for dementia: An updated review. Aust N Z J Psychiatry. 2001;35:776–81 Available from: https://www.ncbi.nlm.nih.gov/pubmed/11990888. 10.1046/j.1440-1614.2001.00967.x 11990888

[131] Panza F, Frisardi V, Capurso C, D’Introno A, Colacicco AM, Imbimbo BP (2010). Late-life depression, mild cognitive impairment, and dementia: possible continuum?. Am J Geriatr Psychiatry.

[132] Kramer AF, Bherer L, Colcombe SJ, Dong W, Greenough WT (2004). Environmental Influences on Cognitive and Brain Plasticity During Aging. J Gerontol Ser A Biol Sci Med Sci.

[133] Kim I-H, Noh S (2014). Ethnic and gender differences in the association between discrimination and depressive symptoms among five immigrant groups. J Immigr Minor Health.

[134] Holmboe-Ottesen G, Wandel M. Changes in dietary habits after migration and consequences for health: a focus on South Asians in Europe. Food Nutr. Res. [Internet]. Co-Action Publishing; 2012 [cited 2016 Jul 13];56. Available from: http://www.ncbi.nlm.nih.gov/pubmed/23139649 10.3402/fnr.v56i0.18891 PMC3492807 23139649

[135] Kim KS, Lee YM, Lee HW, Jacobs DR, Lee DH. Associations between organochlorine pesticides and cognition in U.S. elders: National Health and Nutrition Examination Survey 1999-2002. Environ. Int. [Internet]. Elsevier Ltd; 2015;75:87–92. Available from: 10.1016/j.envint.2014.11.003 10.1016/j.envint.2014.11.003 25461417

[136] Das I, Jagger P, Yeatts K. Biomass Cooking Fuels and Health Outcomes for Women in Malawi. Ecohealth [Internet]. Springer US; 2016; Available from: http://link.springer.com/10.1007/s10393-016-1190-0 10.1007/s10393-016-1190-0 PMC5357447 27800583

[137] Bruce N, Perez-Padilla R, Albalak R (2000). Indoor air pollution in developing countries: a major environmental and public health challenge. Bull World Health Organ World Health Organization.

[138] Fullerton DG, Bruce N, Gordon SB. Indoor air pollution from biomass fuel smoke is a major health concern in the developing world. Trans. R. Soc. Trop. Med. Hyg. [Internet]. Oxford University Press; 2008 [cited 2016 Dec 6];102:843–51. Available from: http://www.ncbi.nlm.nih.gov/pubmed/18639310 10.1016/j.trstmh.2008.05.028 PMC2568866 18639310

[139] Kioumourtzoglou M-A, Schwartz JD, Weisskopf MG, Melly SJ, Wang Y, Dominici F, et al. Long-term PM2.5 Exposure and Neurological Hospital Admissions in the Northeastern United States. Environ. Health Perspect. 2015 124 [cited 2016 Dec 6]. Available from: http://ehp.niehs.nih.gov/1408973 10.1289/ehp.1408973 PMC4710596 25978701

[140] Oudin A, Forsberg B, Adolfsson AN, Lind N, Modig L, Nordin M, et al. Traffic-Related Air Pollution and Dementia Incidence in Northern Sweden: A Longitudinal Study. Environ. Health Perspect. [Internet]. National Institute of Environmental Health Science; 2016 [cited 2018 Jan 31];124:306–12. Available from: http://www.ncbi.nlm.nih.gov/pubmed/26305859 10.1289/ehp.1408322 PMC4786976 26305859

[141] Weuve J, Puett RC, Schwartz J, Yanosky JD, Laden F, Grodstein F. Exposure to Particulate Air Pollution and Cognitive Decline in Older Women. Arch. Intern. Med. [Internet]. American Medical Association; 2012 [cited 2018 Jan 31];172:219. Available from: http://archinte.jamanetwork.com/article.aspx?doi=10.1001/archinternmed.2011.683 10.1001/archinternmed.2011.683 PMC3622279 22332151

[142] Zhang Z, Plassman B, Xu Q, Zahner G, Wu B, Gai M (2009). Lifespan influences on mid- to late-life cognitive function in a Chinese birth cohort. Neurology.

[143] Han W-J, Shibusawa T. Trajectory of physical health, cognitive status, and psychological well-being among Chinese elderly. Arch. Gerontol. Geriatr. [Internet]. Elsevier Ireland Ltd; 2015;60:168–77. Available from: https://www.sciencedirect.com/science/article/pii/S0167494314001526 10.1016/j.archger.2014.09.001 25277446

[144] McEniry M. Early-life conditions and older adult health in low- and middle-income countries: a review. J. Dev. Orig. Health Dis. [Internet]. Cambridge University Press; 2013 [cited 2015 Nov 13];4:10–29. Available from: http://journals.cambridge.org/abstract_S2040174412000499 10.1017/S2040174412000499 PMC3540412 23316272

[145] Melrose RJ, Brewster P, Marquine MJ, MacKay-Brandt A, Reed B, Farias ST (2015). Early life development in a multiethnic sample and the relation to late life cognition. J Gerontol Ser B Psychol Sci Soc Sci.

[146] Zhang Z, Gu D, Hayward MD (2008). Early Life Influences on Cognitive Impairment Among Oldest Old Chinese. J Gerontol Ser B Psychol Sci Soc Sci.

[147] Raikkonen K, Kajantie E, Pesonen A-K, Heinonen K, Alastalo H, Leskinen JT, et al. Early life origins cognitive decline: findings in elderly men in the Helsinki Birth Cohort Study. PLoS One. 2013:8:e54707 Available from: http://www.pubmedcentral.nih.gov/articlerender.fcgi?artid=3559835&tool=pmcentrez&rendertype=abstract. 10.1371/journal.pone.0054707 PMC3559835 23382945

[148] Brayne C (2007). The elephant in the room - healthy brains in later life, epidemiology and public health. Nat Rev Neurosci.

[149] Ferri CP, Prince M, Brayne C, Brodaty H, Fratiglioni L, Ganguli M (2005). Global prevalence of dementia: A Delphi consensus study. Lancet.

[150] Mathuranath PS, Cherian PJ, Mathew R, Kumar S, George A, Alexander A, et al. Dementia in Kerala, South India: Prevalence and influence of age, education and gender. Int J Geriatr Psychiatry [Internet]. 2010 25:290–297 [cited 2017 Jun 18]. Available from: http://www.ncbi.nlm.nih.gov/pubmed/19621355 10.1002/gps.2338 PMC4367932 19621355

[151] Rogaeva E, Meng Y, Lee JH, Gu Y, Kawarai T, Zou F (2007). The neuronal sortilin-related receptor SORL1 is genetically associated with Alzheimer disease. Nat Genet.

[152] Bertram L, McQueen MB, Mullin K, Blacker D, Tanzi RE (2007). Systematic meta-analyses of Alzheimer disease genetic association studies: the AlzGene database. Nat Genet.

[153] Farrer LA, Cupples LA, Haines JL, Hyman B, Kukull WA, Mayeux R (1997). Effects of age, sex, and ethnicity on the association between apolipoprotein E genotype and Alzheimer disease. A meta-analysis. APOE and Alzheimer Disease Meta Analysis Consortium. JAMA.

[154] Sheffler J, Moxley J, Sachs-Ericsson N (2014). Stress, race, and APOE: understanding the interplay of risk factors for changes in cognitive functioning. Aging Ment. Health.

[155] Rosvall L, Rizzuto D, Wang HX, Winblad B, Graff C, Fratiglioni L (2009). APOE-related mortality: Effect of dementia, cardiovascular disease and gender. Neurobiol Aging.

[156] Corbo RM, Scacchi R (1999). Apolipoprotein E (APOE) allele distribution in the world. Is APOE*4 a “thrifty” allele? Ann. Hum. Genet.

[157] Eisenberg DT (2010). a, Kuzawa CW, Hayes MG. Worldwide allele frequencies of the human apolipoprotein E gene: Climate, local adaptations, and evolutionary history. Am J Phys Anthropol.

[158] Kalaria RN, Maestre GE, Arizaga R, Friedland RP, Galasko D, Hall K, et al. Alzheimer’s disease and vascular dementia in developing countries: prevalence, management, and risk factors. Lancet. Neurol. Elsevier; 2008 [cited 2015 Jun 4];7:812–26. Available from: http://www.thelancet.com/article/S1474442208701698/fulltext 10.1016/S1474-4422(08)70169-8 PMC2860610 18667359

[159] Bettens K, Brouwers N, Engelborghs S, De Deyn PP, Van Broeckhoven C, Sleegers K (2008). SORL1 is genetically associated with increased risk for late-onset Alzheimer disease in the Belgian population. Hum Mutat.

[160] Ning M, Yang Y, Zhang Z, Chen Z, Zhao T, Zhang D (2010). Amyloid-β-Related Genes SORL1 and ACE are Genetically Associated With Risk for Late-onset Alzheimer Disease in the Chinese Population. Alzheimer Dis Assoc Disord.

